# Protein arginylation regulates cellular stress response by stabilizing HSP70 and HSP40 transcripts

**DOI:** 10.1038/cddiscovery.2016.74

**Published:** 2016-10-03

**Authors:** Kamalakshi Deka, Archana Singh, Surajit Chakraborty, Rupak Mukhopadhyay, Sougata Saha

**Affiliations:** 1 Department of Molecular Biology and Biotechnology, Tezpur University, Napaam, Assam, India

## Abstract

ATE1-mediated post-translational addition of arginine to a protein has been shown to regulate activity, interaction, and stability of the protein substrates. Arginylation has been linked to many different stress conditions, namely ER stress, cytosolic misfolded protein stress, and nitrosative stress. However, clear understanding about the effect of arginylation in cellular stress responses is yet to emerge. In this study, we investigated the role of arginylation in heat-stress response. Our findings suggest that Ate1 knock out (KO) cells are more susceptible to heat stress compared with its wild-type counterparts due to the induction of apoptosis in KO cells. Gene expression analysis of inducible heat-shock proteins (HSP70.1, HSP70.3, and HSP40) showed induction of these genes in KO cells early in the heat shock, but were drastically diminished at the later period of heat shock. Further analysis revealed that loss of ATE1 drastically reduced the stability of all three HSP mRNAs. These phenotypes were greatly restored by overexpression of Ate1 in KO cells. Our findings show that arginylation plays a protective role during heat stress by regulating HSP gene expression and mRNA stability.

## Introduction

Protein arginylation is a global regulator of cellular function and tissue development.^1^ This emerging protein modification has been shown to affect cellular function by regulating protein activity, interaction, and stability.^2–5^ Homozygous loss of Ate1, the gene responsible for arginylation, causes embryonic lethality due to defect in cardiovascular development, angiogenesis, and neural tube development.^6,7^ Tissue or cell type specific loss of Ate1 causes defective heart muscle development, neural crest morphogenesis, and spermatogenesis.^8–10^ Whole body deletion of Ate1 after birth increased metabolic rate and caused defect in nervous system and spermatogenesis.^11^ A large variety of proteins identified as target for arginylation shown to regulate many of the cell’s function, including cell migration, cell proliferation, tumorigenesis, apoptosis, actin cytoskeletal dynamics, cell-to-cell adhesion, purine metabolism, G-protein signaling, oxidative stress sensing, and stress response.^2,12–20^ Among these stress response is focus of the current investigation.

Stress response at the cellular level is primarily a defense reaction towards any stress conditions and thus a potent stress response is crucial for cell survivability and recovery. However, if the stress condition is unresolved, cell induces yet another pathway that is opposite to cell survival, that is, cell death. It has been shown that these two pathways functionally interact at many places to determine the cell fate in a stress condition.^21^ Heat-shock proteins (HSPs) play important roles in integration of these two pathways. HSPs are part of minimal set of proteins involved in stress response and are highly conserved from bacteria to mammals.^22,23^ Different HSPs, mostly involved in protein folding, impart a protective effect to stress condition by stabilizing protein structure and function. In addition to their protein folding function, a large number of studies reported that a part of HSPs’ protective function is due to its inhibitory effect at different steps in apoptosis pathway.^24–26^

Post-translational protein arginylation has been linked to many cellular stress conditions, namely ER stress, cytosolic misfolded protein stress, and nitrosative stress. It has been shown that arginylation is sensor of NO and responds to nitrosative stress through arginylation of oxidized Cys residues.^17^ Several ER-resident proteins are shown to be substrate of arginylation. Arginylation of calreticulin, an ER-resident chaperon, is induced by heat shock and few other stresses.^18^ This modification of calreticulin is important for its dimerization and localization in stress granule that helps in scaffolding of large stress granules. Thus, lack of arginylation impaired formation of stress granules that help protect cellular RNA during stress conditions.^27^ Arginylated calreticulin is also suggested as pre-apoptotic signal as calreticulin of Ate1 knock out (KO) cells found to be resistant to ER stress inducing arsenite treatment.^28^ Another ER-resident chaperon and HSP70 group of protein, GRP78, found to be arginylated during cytosolic misfolded protein stress.^29^ N-terminal arginylation of GRP78 induces its interaction with autophagic adapter p62 in cytosol leading to its oligomerization and interaction with autophagosome component LC3. Several other molecular chaperons, namely, chaperonin, HSPA8, Ribophorin I, HSP90*β*, and HSP90*α* also found to be arginylated.^30,31^ Although HSPA8 (HSP70) and HSP90*β* are constitutively expressed HSPs, HSP90*α* is induced upon heat shock and the post translationally added arginine on this protein is further modified by methylation.^31^ Mounting evidence suggest a broader role of arginylation in regulation of these groups of proteins. However, further study is required to fully understand this phenomena.

In this study using an Ate1 KO genetic model and heat-shock stress model, we addressed the question: what is the role of arginylation in heat-stress response? Our findings suggest that Ate1 KO mouse embryonic fibroblasts (MEFs; KO cells) are more susceptible to heat stress compared with its wild-type (WT) counterparts, a phenotype that can be rescued by stable expression of Ate1 in KO MEFs. Although at the given heat-stress condition WT MEFs were protected, apoptosis was induced in KO MEFs. Gene expression analysis of inducible HSPs, HSP70.1, HSP70.3, and HSP40 showed induction in KO MEFs during shorter period of heat shock. However expressions of these genes are drastically diminished in the KO MEFs upon longer period of heat shock that got reverted by expression of Ate1. Further analysis suggested that loss of arginylation (Ate1 KO) drastically reduced the stability of all three HSP mRNAs that can be reverted by overexpression of Ate1. Our findings suggest that arginylation plays a protective role during heat-stress response by regulating HSP gene expression and mRNA stability.

## Results

### Loss of arginylation makes cells more susceptible to heat stress

**Figure 1:**
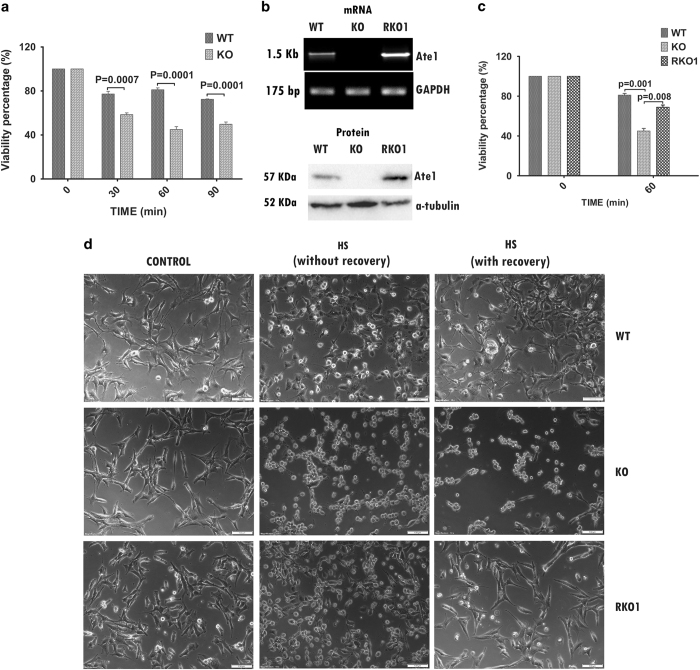
Loss of arginylation increased susceptibility of MEFs to heat stress. (**a**) Viability percentage of WT and KO MEFs (KO) after heat shock at 44 °C for different durations (0, 30, 60, and 90 min) followed by recovery for 8 h at 37 °C, (*n=*4). (**b**) RT–PCR and western blot results confirming the stable transfection and overexpression of Ate1-1 into KO MEF cells (RKO1). (**c**) Viability percentage of WT, KO, and RKO1 cells after heat shock at 44 °C for 60 min followed by recovery for 8 h at 37 °C, (*n=*4). (**d**) Phase-contrast micrographs of WT, KO, and RKO1 MEFs from experiment described in **c**. Control: cell incubated at 37 °C; HS, heat shock. Data represent mean±s.e.m. *P*-values: Student's *t*-test.

Earlier reports linked arginylation to many stress responses and KO cells are found to be resistant to arsenite-induced ER stress.^28^ To test the role or arginylation during heat-stress response, WT and KO MEFs were subjected to heat shock at different temperature and duration. To determine the temperature at which significant heat stress is induced in these cells as evident by loss of cell viability, cells were exposed to four different temperatures (37, 40, 42, and 44 °C) for 90 min. As reported in earlier studies, it was found that significant stress is induced at 44 °C in these cells (Supplementary Figure S1).^32^ Thus, for all the subsequent experiments 44 °C was used as heat-shock temperature. WT and KO cells were exposed to heat stress for 30, 60, and 90 min followed by recovery at 37 °C for 8 h. It was observed that at all the time points KO cells have reduced viability compared with WT cells (Figure 1a). To determine whether reduction in viability of KO cells upon heat stress is due to loss of arginylation, we attempted to recover this phenotype by stable overexpression of one of the ATE1 isoforms ATE1-1 that is expressed in high amount in WT cells, (Supplementary Figure S2).^33,34^ Recombinant cells, henceforth termed as RKO1, showed high-level expression of Ate1-1 transcript and protein (Figure 1b). Exposure of RKO1 cells and other cell lines to heat stress showed increased survivability of the RKO1 cells compared with KO cells (Figure 1c). Even though survivability percentage of RKO1 cells was somewhat less compared with WT cells, the extent of recovery was quite high. Microscopic observation of three cell types immediately after heat stress without recovery and after recovery at 37 °C showed that stress caused deformities in all three types of cells immediately after heat shock. However, WT and RKO1 cell recovered after incubation at 37 °C, but majority of the KO cells failed to do so (Figure 1d). Therefore, the high degree of protection of cells from heat-shock treatment appears to be dependent upon the expression of Ate1 gene.

### Stable overexpression of Ate1.1 isoform protects MEFs from heat-shock-induced apoptosis

**Figure 2:**
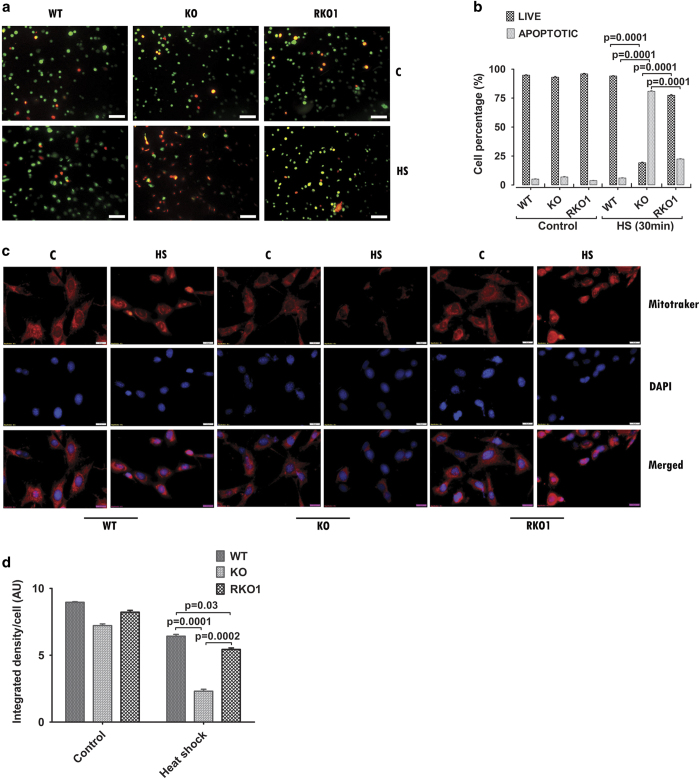
Loss of arginylation increased apoptosis in MEFs upon heat stress. (**a**) Fluorescence micrographs of acridine orange/ethidium bromide (AO/EB) stained cells after heat shock at 44 °C for 30 min followed by recovery for 8 h at 37 °C (HS). Control cells (C) were incubated at 37 °C. Scale bars, 100 μm. (**b**) Graph represents of percentage of apoptotic (orange/red) and live (green/light yellow) cells in control (C) and heat-shocked (HS) cells in the experiment described in **a**. Data represent average of four frames. Total number of cells counted for analysis were WT: 486 (C), 511(HS), KO: 487(C), 507 (HS), and RKO1: 523(C), 426 (HS). (**c**) Fluorescence micrographs of mitochondrial membrane potential-dependent mitochondrial dye, MitoTracker Orange CMTMRos (300 nM), stained cells after heat shock (HS) at 44 °C for 30 min followed by recovery of 6 h at 37 °C. Control (C) cell were incubated at 37 °C. Nuclei were stained with DAPI. Scale bars, 20 μm. (**d**) Graph represents quantification of the data described in **c**. Average ‘integrated fluorescence density’ per cell in control and heat-shock conditions are plotted for WT, KO, and RKO1 cells. Data represent average of four frames. Total number of cells counted for analysis were, WT: 18 (C), 38 (HS), KO: 38(C), 43(HS), and RKO1: 27(C), 38(HS). Data represent mean±s.e.m. *P*-values: Student's *t*-test.

Cell survivability and cell death walks hand in hand during a stress condition and it has been shown that improper resolution of heat-stress condition due to poor stress response leads to the activation of cell death pathways.^24–26^ To test the apoptotic status of Ate1 KO cells upon heat shock and the contribution of arginylation in that process, percentage of apoptotic cells were determined in heat-stressed WT, KO, and RKO1 MEFs. Such analysis clearly showed that, although >60% of heat-stressed KO cells were in apoptotic phase, <10% of the heat-stressed WT cells were undergoing apoptosis. Stable overexpression of ATE1-1 isoform (RKO1 cells) showed recovery from heat-shock-induced apoptosis with >70% of cells present in live phase following heat shock (Figures 2a and b). As mounting evidences suggest that HSPs have regulatory role in both intrinsic and extrinsic pathways^35^ of apoptosis and in most of the cases apoptosis induced due to unresolved heat-stress are associate with mitochondrial dysfunction and downstream pathways,^24,26,36–38^ mitochondrial integrity in heat-stressed WT, KO, and RKO1 cells were tested by mitochondrial membrane potential-dependent accumulation of MitoTracker Orange CMTMRos. It was observed that although all three cell types at 37 °C have comparable levels of mitochondrial staining, in heat-stress condition KO cells contained much less mitochondrial stain compared with WT and RKO1 MEFs (Figures 2c and d). These data suggested that heat stress induced severe mitochondrial dysfunction in absence of arginylation. Hence, presence of Ate1 gene is very much instrumental in activating better stress response machinery in stress condition, allowing cells to diminish the activation of programmed cell death cascade and making them resistant to heat-shock-induced apoptosis.

### ATE1 renders cell viability upon heat stress via regulating expression of HSP genes

**Figure 3:**
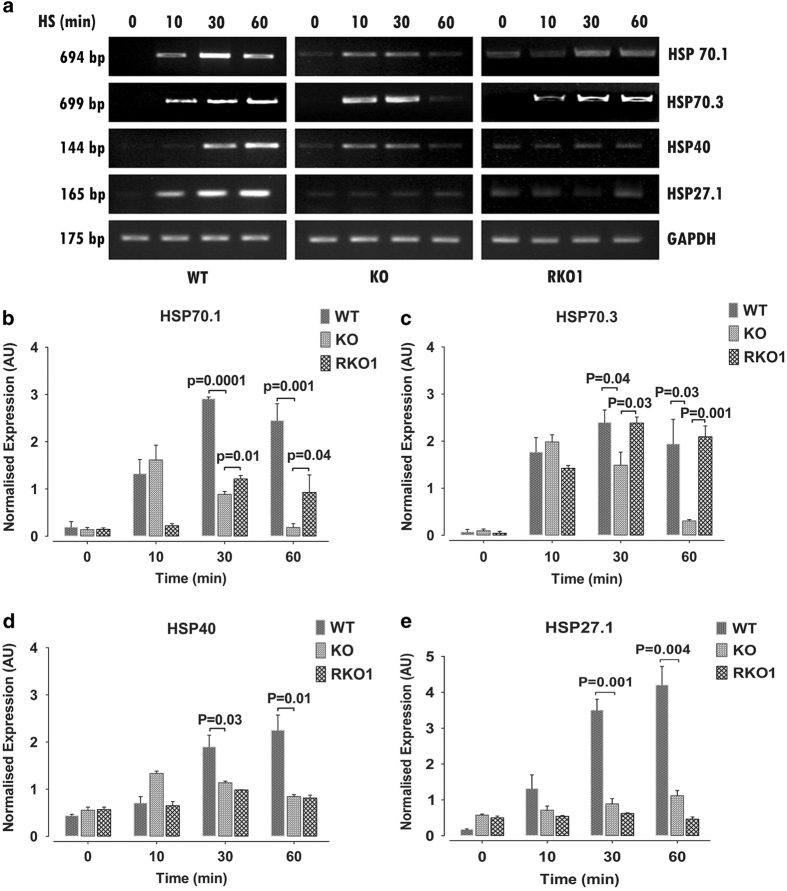
ATE1 regulates gene expression of important heat-shock proteins during heat stress. (**a**) Representative gel images from RT–PCR analysis of four major inducible heat-shock genes (HSP70.1, HSP70.3, HSP40, and HSP27) in WT, KO, and RKO1 cells after exposing to heat-shock (HS) at 44 °C for different time duration followed by recovery for 6 h at 37 °C. (**b**–**e**) Normalized expression level of different HSPs in WT, KO, and RKO1 cells following heat stress as described in **a**. Gene expressions were normalized to GAPDH, (*n=*3). Data represent mean±s.e.m. *P*-values: Student's *t*-test.

Heat shock induces a large variety of proteins, including several classes of HSPs that play a major role in stress response by sensing macromolecule damage, and stabilizing and refolding of misfolded proteins. Apart from this classical function of HSPs, they also play an important role in inhibition of apoptosis that helps cells to recover from stress condition. Three of the HSPs, HSP70, its co-chaperon HSP40, and HSP27 are found to be directly involved in maintaining mitochondrial membrane integrity during heat shock.^35^ To find out what is causing KO cells to become more sensitive to heat shock and lose its ability to activate its stress response machinery during recovery from stress, we checked the expression profile of stress marker genes HSP70.1, HSP70.3, HSP40, and HSP27.1 after exposing cells to heat stress for different time period followed by recovery at 37 °C. Gene expression analysis showed that although all four genes were induced in WT cells, KO cells showed induction of HSP70.1, HSP70.3, and HSP40, but not HSP27 (Figures 3a–e). However, an intriguing fact observed with HSP70.1, HSP70.3, and HSP40 induction in KO cells was the induction of gene expressions were comparable to WT cells during early period of heat-shock treatment, but failed to sustain during longer period of heat stress (Figures 3a–d). This suggested that transcriptional activation in these gene promoters are not affected in the absence of arginylation and loss of gene expression at longer period of heat stress is due to some other reason. The dependency of HSP gene expressions on presence of ATE1 was further confirmed with recovery of HSP70.1 and HSP70.3 expression in RKO1 cells during heat shock (Figures 3a–c). Expression of HSP40 was also somewhat recovered in RKO1 cells due to the fact that its expression is sustained during late in the heat-stress treatment, but with much lower level. Overexpression of ATE1-1 could not rescue HSP27.1 expression in RKO1 cells (Figures 3a and e).

### ATE1 regulates stability of HSP70.1, HSP70.3, and HSP40 transcripts

**Figure 4:**
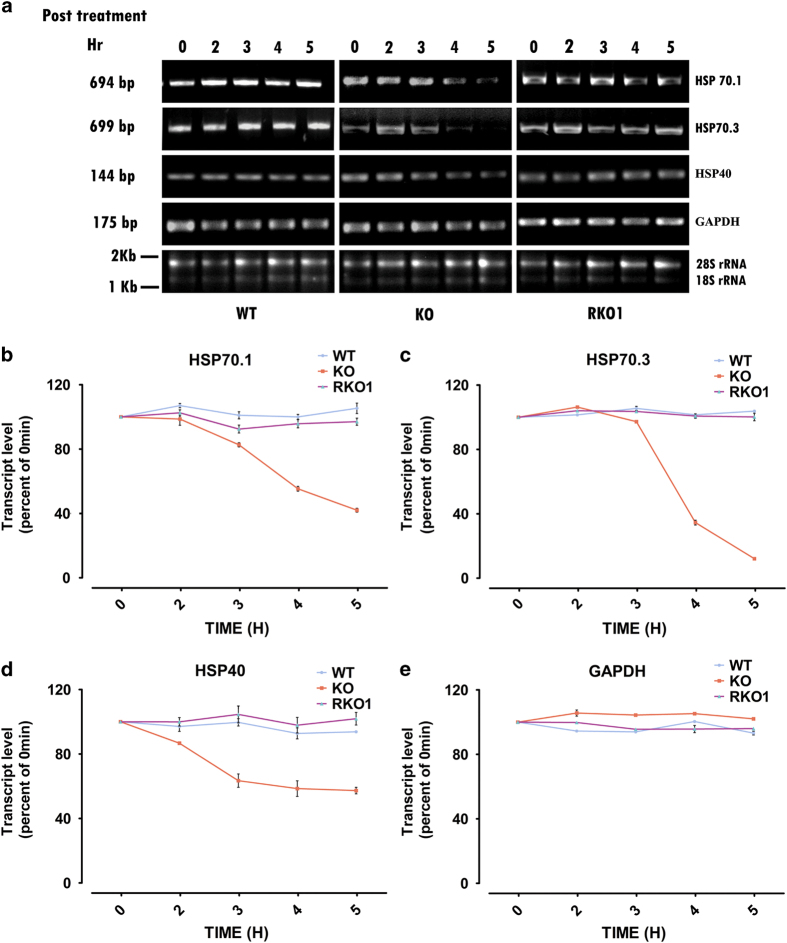
ATE1 regulates stability of HSP transcripts. (**a**) Representative gel images from RT–PCR analysis for three heat-shock genes (HSP70.1, HSP70.3, and HSP40) and a housekeeping gene (GAPDH) in heat-stressed WT, KO, and RKO1 MEFs at different time points (0–5 h) post actinomycin D (5 *µ*g/ml) treatment to inhibit RNA synthesis. (**b**–**e**) Graphs representing the normalized transcript levels for HSPs and GAPDH in actinomycin D-treated cells as described in **a**. Transcript levels were normalized by calculating ratio of gene of interest/18S rRNA for each sample and taking 0 min as 100%, (*n=*3). Data represent mean±s.e.m. *P*-values: Student's *t*-test.

Observed gene expression pattern of these three HSP genes in KO cells clearly suggested that transcriptional activation of these genes did happen in these cells during early stage of heat-shock response, but lost during later period of heat-shock treatment. Earlier reports showed that HSP70 mRNA is stabilized during heat-shock treatment and found to have much higher stability compared with non-stress condition.^39^ We tested whether loss of ATE1 has any effect on HSP mRNA stability. It was observed that all three HSP transcripts were stabilized during heat stress in WT cells (Figure 4). However, HSP transcripts showed different degree of instability in KO cells after heat stress. Among three HSP genes, HSP70.3 was most susceptible and its level reached <10% by 5 h post inhibition of fresh transcription (Figure 4c). Interestingly, loss of ATE1 did not affect the stability of housekeeping gene transcript as GAPDH transcript is found to be quite stable in KO cells post heat stress (Figure 4e). Failures of stabilization of three HSP transcripts were recovered by ATE1-1 overexpression in RKO1 cells. The mRNAs of all three HSP genes showed stabilization in RKO1 cells after heat-shock treatment (Figures 4b–d).

## Discussion

Arginylation has been linked to multiple cellular stress responses.^17,18,29^ Current study showed that arginylation plays an important role in heat-stress response. The fact that KO MEFs have reduced survivability rate upon heat stress (Figure 1a) suggested that arginylation is important to mount a potent stress response rendering protection to the cells in stress conditions. In support of this notion, it was observed that arginylation regulates gene expression of major classes of HSP proteins (HSP70, HSP40, and HSP27) that are part of the minimal stress response gene induced in different stress conditions (Figure 3). An earlier study has shown that these cells are more resistant to arsenite-induced ER stress conditions due to inhibition of apoptosis. Interestingly, we observed that these cells are more susceptible to heat-stress condition due to induction of apoptosis. Yet another study showed that arginylation has an anti-apoptotic function due to arginylation-dependent degradation of many pro-apoptotic factors. Exposure to UV radiation induced higher level of apoptosis in KO cells compared with WT cells.^20^ These different phenotypes could be due to different role played by arginylation at different stress conditions by affecting different target proteins and pathways. During stressful conditions cell responds via some general stress response pathways and some stressor specific response pathways.^23^ Arginylation being a global regulator with multiple known targets, it possibly has diverse effect during stress responses from rendering protection in one stress condition to detrimental effect in another. Moreover, apoptosis induced due to non-resolution of heat stress may induce extrinsic, as well as intrinsic pathways. During heat-stress loss of mitochondrial integrity in KO cells indicated involvement of intrinsic pathway mediated apoptosis in these cells (Figure 2b). However, this does not exclude the role of extrinsic pathway in this process, and further investigation is required to elucidate the exact mechanism of apoptosis in these cells and its relation to arginylation.

Although it was clear that the loss of arginylation made MEFs more susceptible to heat stress that could be reverted by expression of ATE1-1, relative role of other arginylation isoforms in this process need to be established.^40^ This is evident from the fact that, even though ATE1-1 expressing RKO1 cells have improved survivability, it is somewhat less than WT cells suggesting possible role of other ATE1 isoforms in the process (Figure 1c). Though the expression of HSP70.1, HSP70.3, and HSP40 was restored in RKO1 cells, ATE1-1 could not restore HSP27 expression (Figure 3e). This also suggested that other isoforms of ATE1 may play important role in rendering protection to the cells in stressful condition by regulating gene expression of HSP27 and possibly many other stress response genes.

The stress response genes whose expressions were found to be affected by loss of arginylation are known to play very important role in functional interaction of stress response and cell death pathways.^21^ Among them, gene expression pattern of two HSP70 isoforms along with HSP40 in heat-stressed KO cells were intriguing due to the fact that expression of these genes which were comparable to WT cells during early period of heat-shock treatment failed to sustain during longer period of heat stress (Figures 3a–d). This phenotype suggested two possibilities: (1) altered promoter activity of these genes in absence of ATE1; and (2) altered stability of these transcripts in absence of ATE1. The fact that these genes are initially induced as expected upon heat stress ascertain that, at least the initial induction circuit working at these promoters are not affected in absence of ATE1. Increased stability of HSP70 transcript upon heat stress has been reported to be important for potent stress response.^39^ This led us to investigate the stability of these transcripts upon heat stress in KO cells. It was observed that arginylation has positive role in stabilizing these transcripts during heat shock, as continuous decay of these transcripts were observed in heat-stressed KO cells (Figure 4). As loss of arginylation has no effect on GAPDH transcript stability, it indicated that, mRNA stabilization effect of arginylation is not a general effect, rather specific to a set of mRNAs. HSP70 and HSP40 has been shown to inhibit the intrinsic pathway of apoptosis by inhibiting Bax, release of cytochrome *c* and assembly of apoptosomes.^24,26,36–38^ Loss of mitochondrial integrity in KO cells during heat-stress conditions suggested activation of the intrinsic pathway of apoptosis. Thus, a possible explanation of reduced viability of KO cells upon heat stress could be poor stress response due to loss of HSP proteins leading to mitochondrial destabilization and induction of apoptosis.

Though it is clear that arginylation is positively affecting stabilization process of HSP mRNAs, the ATE1 target protein in this pathway remains to be identified. Earlier studies have shown that protein eEF1A1 and micro RNA miR-378* directly regulate HSP70 mRNA stability. Direct interaction of eEF1A1 with 3′ UTR of HSP70 mRNA stabilizes this transcript and facilitates its nuclear export.^41^ Although miR-378* supposed to degrade HSP70 mRNA, oxidative stress, and heat stress induces HSP70 transcripts with shorter 3′UTR lacking miR-378* target site is unsusceptible to micro RNA mediated degradation.^42^ Three other miRNAs, miRNA-1, miRNA-21, and miRNA-24 were upregulated during ischemic conditions reported to increase HSP70 mRNA level.^43^ However, it is not clear whether the effects of these miRNAs are via regulation of HSP70 gene expression or stability of its mRNA. None of these pathways are reported to be target of ATE1. It remains to be investigated whether stabilizing effect of HSP transcripts by arginylation is mediated by regulation of any of these pathways or some other yet to be identified novel pathway.

In conclusion, here we report that, post-translational protein arginylation that is known for regulation of protein stability and function has an important role in cellular stress response mediated by yet another unique function that is regulating mRNA stability of HSPs.

## Materials and Methods

### Cell culture and heat-shock treatments

MEFs (kind gift from Dr Anna Kashina, University of Pennsylvania) were grown in complete medium containing DMEM/F10 supplemented with 10% FBS and 1× antibiotic/antimycotic. For induction of stress, cells were incubated at 44 °C for different time period followed by recovery of 6–8 h (as per different experiments) at 37 °C. Control cells were kept at 37 °C for entire duration of the experiment.

### Cell viability assay

To study cell viability, 5000 cells per well of a 96 well plates were seeded. Cells were grown in presence of 5% CO_2_ at 37 °C overnight. On reaching confluency of 50–60%, cells were heat stressed for different duration followed by recovery at 37 °C for 8 h with control cells kept at 37 °C throughout. Post recovery, viability test was done using MTT assay (Sigma Aldrich M2128, St Louis, MO, USA).

### Construction of pMSCV-PIG-Ate1-1 clone and preparation of recovered cell line

Ate1-1 ORF was PCR amplified using a forward primer (5′ -ATACTCGAGGCCGCCACCATGG CTTCTTGGAGCGCGCCTTCA-3′) and a reverse primer (5′ -ATAGTTAACTCACATCATCAT CATCATCATGTGTCTGAACAGCAGCATCCTCTCCGA-3′) from an existing clone of Ate1-1 (kind gift from Dr Anna Kashina, University of Pennsylvania).^33^ Amplified Ate1-1 ORF was cloned into a modified retroviral pMSCV vector, pMSCV PIG (Addgene plasmid 21654, Cambridge, MA, USA),^44^ using XhoI (NEB R0146S, Ipswich, MA, USA) and HpaI (NEB R0105S; Supplementary Figure S2). Lack of mutation was confirmed by sequencing with both forward and reverse primers. This construct was stably transfected in KO MEFs for preparation of recovered cell line. To generate virus like particles, HEK 293T cells were transfected with Ate1-1 construct along with two other plasmids containing retroviral GAG-pol, and Env gene using lipofectamine reagent (Invitrogen, Waltham, MA, USA; cat # 11668-027). Finally to prepare cell line with stable expression of Ate1-1 isoform, KO MEF cells were transfected with generated virus like particles containing Ate1-1 construct in the presence of polybrene (Sigma Aldrich 107689). Positive transfectants were selected in presence of puromycin (20, 40 and 60* µ*g/ml; Merck 540411, Kenilworth, NJ, USA) for several generations. Confirmation of stable expression was done by RT–PCR and western blot analysis using a monoclonal ATE1 antibody (clone 6F11, kind gift from Dr Anna Kashina) after removing the cells from puromycin selection pressure. These cells were named as RKO1.

### Apoptosis sssay (AO/EtBr method)

For apoptosis assay, 3×10^5^ cells were seeded into 35 mm culture dish and incubated in 5% CO_2_ at 37 °C overnight. On reaching a confluency of 60–70%, cells were heat stressed for 30min followed by recovery for 8 h at 37 °C with controls cells kept at 37 °C. Post recovery, entire cell population including floating cells were collected in a tube and stained with 1:1 ratio of acridine orange and ethidium bromide at a concentration of 100 *μ*g/ml each. Following 3–5 min of incubation, 20 *μ*l of the suspension was mounted on glass slides and studied under a fluorescence microscope using 40×objective and RFP/TRITC filter (Leica filter series N2.1, Wetzlar, Germany; cat # 11513882).^45^ For every cell type, all cells in four image frames were analyzed using ImageJ software (NIH, Bethesda, MD, USA). Cells stained green and light yellow were counted as live cells and cells stained orange/red were counted as apoptotic cells.

### Mitochondrial staining

A total of 5×10^4^ cells were seeded and grown on cover slip in a 35 mm culture dish. On reaching confluency of ~40–50%, cells were heat stressed for 30 min followed by recovery of 6 h at 37 °C. Cell incubated at 37 °C was taken as control. Following recovery cells were further incubated with 300 nM concentration of MitoTracker Orange CMTMRos (Invitrogen M7510) dissolved in DMEM (w/o FBS) for 30 min at 37 °C. Cells were washed with pre-warmed PBS and fixed using 4% paraformaldehyde in PBS (Himedia, Chennai, India) for 15 min. After fixation, cells were rinsed several times in PBS and incubated in permeabilization buffer containing 0.2% Triton X-100 for 10 min. Cells were washed with PBS thrice and counterstained with DAPI for 10 min at room temperature. Finally, coverslips were mounted onto a microscopic glass slide using ProLong Gold antifade reagent (Invitrogen P10144) and viewed under fluorescence microscope using 40× objective and a RFP/TRITC filter. Images were further analyzed using ImageJ software and ‘integrated density value’ per cell was plotted. Total of four frames were taken for analysis.

### Gene expression analysis

A total of 5×10^5^ cells were seeded onto 35 mm culture dish and incubated in 5% CO_2_ at 37 °C for overnight. On reaching confluency of ~70%, cells were heat stressed for different durations followed by recovery of 6 h. Cells incubated at 37 °C were taken as control. Following recovery, cells were harvested for total RNA isolation using TRizol reagent (Invitrogen) and cDNA was prepared using a cDNA synthesis kit (Clontech, Otsu, Japan; 6110A), following the manufacturers’ protocol. Semi quantitative RT–PCR was performed for four of the selected heat-shock marker genes (HSP70.1, HSP70.3, HSP40, and HSP27.1) using following primers: HSP70.1 (forward—5′ -CACCACCTACTCGGACAACC-3′; reverse—5′ -GCAAAGAGTC TGTTTTCTAGACCA-3′), HSP70.3 (forward—5′- CGCAGACCTTCACCACCTAC-3′; reverse—5′- CCACATATCTGTCTCCTAGCCAG-3′), HSP40 (forward—5′- TGAGGCTACTCTGGACG AAAG-3′; reverse—5′ -GGAGGCACACGCTCAAATAAA-3′), HSP27.1 (forward—5′- GGGTGC AGGTTGCTCTTAAA-3′; reverse—5′- CAGAAGGTGTGTTGGGTCTTC-3′). GAPDH (forward—5′- CGACTTCAACAGCAACTCCCACTCTTCC-3′; reverse—5′ -TGGGTGGTCCAGGGTTTCTT ACTCCTT-3′) was taken as internal control. Electrophorogram of RT–PCR products were quantified by densitometry analysis of gel pictures using GelQuant.NET software (Biochemlabsolutions, Wayne, PA, USA) provided by biochemlabsolutions.com and normalized by calculating band signal ratio of gene of interest/GAPDH.

### mRNA decay assay

mRNA stability was analyzed following the method described by Theodorakis and Morimoto^39^ with some modifications. A total of 5×10^5^ cells were seeded into 35 mm culture dish and incubated in 5% CO_2_ at 37 °C overnight. On reaching confluency of ~70%, cells were heat stressed for 20 min and kept for recovery of 2 h at 37°C before addition of actinomycin D at a concentration of 5* µ*g/ml. Plates were collected for total RNA isolation at 0, 2, 3, 4, and 5 h after addition of actinomycin D. RNA was isolated using Trizol reagent (Invitrogen) following manufacturer’s protocol. RT–PCR was done for HSP70.1, HSP70.3, HSP40, and GAPDH. Electrophorogram of RT–PCR products were quantified using GelQuant.NET software provided by biochemlabsolutions.com and normalized by calculating band signal ratio of gene of interest/18S rRNA for each sample.

### Statistical analysis

For all the experiments, error bar is s.e.m. and unpaired Student *t*-test was done to calculate the *P*-values using graph pad prism 5.0 (Graph Pad software, La Jolla, CA, USA).
