# Supplementary figures and images for: Protein arginylation regulates cellular stress response by stabilizing HSP70 and HSP40 transcripts
Source: Cell Death Discov. 2016 Oct 3;2:16074–. doi: 10.1038/cddiscovery.2016.74 (PMC5045964; doi:10.1038/cddiscovery.2016.74)

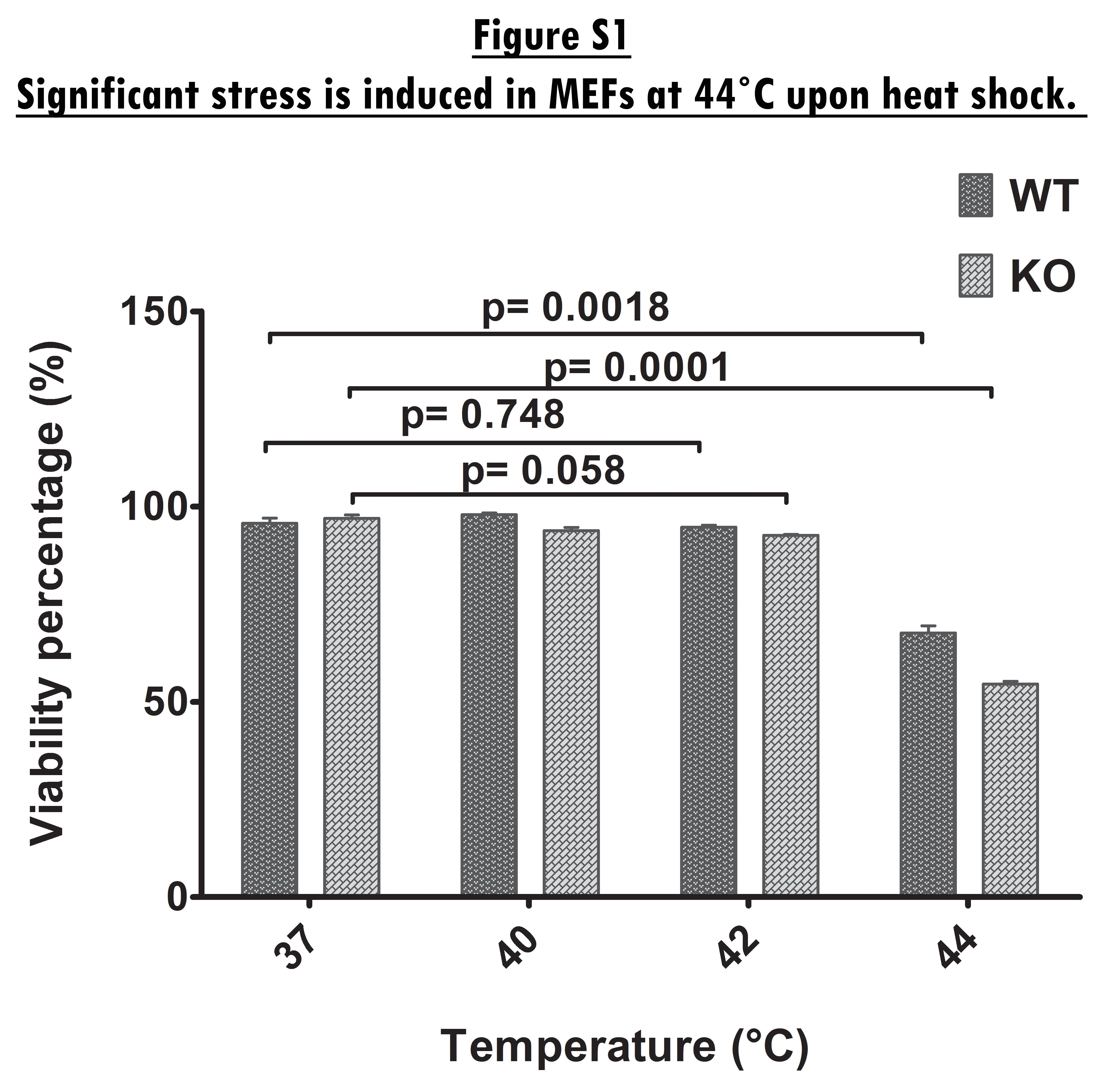

Supplement: Supplementary Figure 1 [file cddiscovery201674-s1.jpg]

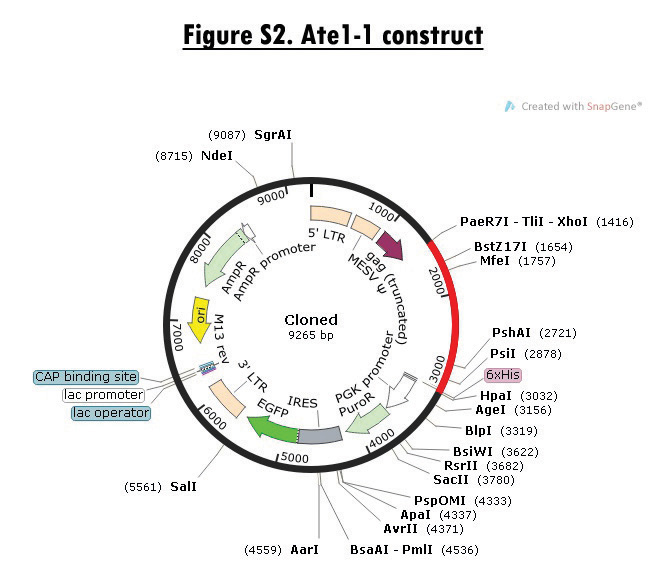

Supplement: Supplementary Figure 2 [file cddiscovery201674-s2.jpg]
